# Analytical study and real simulation for improving the safety of ageing nuclear facility using UPFC

**DOI:** 10.1038/s41598-023-50356-1

**Published:** 2024-01-02

**Authors:** Ahmed S. Adail, Yasser M. Ammar, Adel A. Elbaset, Sayed EL. Araby

**Affiliations:** 1 Nuclear Fuel Technology Department, Hot Labs Center, Egyptian Atomic Energy Authority (EAEA), Inshas, Egypt; 2 Egypt Second Research Reactor Complex, Egyptian Atomic Energy Authority (EAEA), Inshas, Egypt; 3 Electrical Engineering Department, Faculty of Engineering, Minia University, El Minia, Egypt; 4 Department of Electro Mechanics Engineering, Faculty of Engineering, Heliopolis University, Cairo, Egypt; 5 Reactors Department, Egyptian Atomic Energy Authority (EAEA), Inshas, Egypt

**Keywords:** Engineering, Mathematics and computing

## Abstract

This paper aims to increase the performance and improve the safety of an ageing Nuclear Facility (NF). Good power quality extends the life of electrical equipment at NF and thus protects it from premature aging. The first stage of this paper presents a measurement and analysis of various power quality events for a real-world case of a NF under different conditions of operation. In the previous work for this group, a new proposed technique based on partial swarm optimization is presented to find the allocation of the UPFC to enhance the power quality within the specified limit. The technique is tested by IEEE33 bus. This step is to assess system performance and find the best solutions to ensure the normal and safe operation of NF. In this paper, the simulink-matlab programme was used to simulate a real NF based on a new vision of UPFC. The results indicate that the strategy is an effective way to improve the safety of power quality and ageing NF using UPFC.

## Introduction

The NF has very sensitive electrical loads affected by disturbances that occur in additional facilities linked to the same feeder through a similar connection point. The NRR consists of the Research Reactor (RR), the Fuel Factory (FF), the Radioisotope Product Facility (RPF), Laboratories Bulding (LB), and infra-structure building^1^.

The power quality (PQ) is affected by the equipment that is operated or connected electrically^2–4^. The classification of these power quality issues according to their definition and the power quality standards is illustrated in^4–6^. Power quality issues can hasten the ageing of electrical equipment. This is due to the fact that these issues can put strain on the insulation and other components of the equipment, causing premature wear and tear. The impact of power quality issues on ageing can be cumulative. Even modest issues might lead to the premature breakdown of electrical equipment over time. Some of the most prevalent power quality issues that might lead to ageing include:

- **Voltage sags and swells**: These are brief changes in voltage that can cause equipment to malfunction or fail.
- **Transients** are unexpected spikes in voltage that can harm sensitive electronic equipment.
- **Harmonic distortion** is a distortion of the sine wave form of the power supply that can cause overheating and other issues with electrical components.

In addition to these frequent concerns, there are a variety of other power quality issues that can contribute to ageing, such as:

- **Grounding issues:** These can generate electrical noise and interference, which can harm sensitive electronics.
- **Power interruptions:** These are total power outages that can cause equipment to shut down and data loss.

A variety of variables can worsen the impact of power quality concerns on ageing, including:

- **The equipment's age and condition**: Older equipment is more likely to be harmed by power quality issues than newer equipment.
- **The operating environment of the equipment**: Power quality issues are more likely to damage equipment that is used in extreme settings, such as high temperatures or humidity.
- **The type of equipment:** Power quality concerns are more likely to harm sensitive electronic equipment than other types of equipment.

Poor power quality raises operational expenses and reduces the usefulness of a system component. Any type of disruption causes the reactor to shut down and incur financial losses. The financial loss depends on the cost and condition of the reactor fuel. The reactor normally takes approximately an hour to restart the following scram, but it takes around 50 h to restart if it is towards the end of the fuel cycle due to bad PQ. From experience, the cost of an NRRF perturbation is about $30,000 in the standard situation. The NRR is affected by poor power quality, like^7^:

- A power failure, which caused the reactor to shut down.
- Failures that cause the reactor to be shut down.
- Some components, such as electrical cards and capacitors, fail.
- Tripping circuit breakers and wreaking havoc on bus ducts.
- Low-load overheating, noise, and transformer non-performance.
- Burning motor coils, overheating, vibration, and noise.
- A disproportionately large percentage of lights and ballasts fail.
- Inconsistent critical system performance.
- Insufficient electrical network dependability.

The Fluke 435-II Power Quality Analyzer is used to measure and analyse PQ results and how they are impacted by the presence of non-linear loads through measurements made on the NRR electrical grid. The NRRF under examination has been in operation for over 25 years, and numerous of its components and equipment are aging. The introduction of FACTS devices introduces new approaches for controlling electricity and improving the utility of distribution systems. FACTS controllers are power electronics-based controllers designed to improve system stability, voltage profile (VP), power flow, and power losses^8,9^.

This paper aims to increase the performance of the electrical grid supply NF by using FACTS devices to improve the voltage sag and swell and minimise the active and reactive power losses of the system. In this regard, the Unified Power Flow Controller (UPFC), being a type of FACTS, is simulated via the Particle Swarm Optimization (PSO) to detect the allocation of UPFC devices in order to achieve the fitness function (the objective function). The suggested approach code for the IEEE standard bus system has been verified in^1^ before being applied to a mini-model of the NFs.

The paper is divided into two sections: The main purpose of this paper is to investigate the manner in which poor power quality can affect NRR by using the Fluke 435-II Power Quality Analyzer. Harmonics and any disturbance, such as active and reactive power loss, voltage swell, and voltage sag, can reflect poor power quality in the electrical system of NRR. The final section is applied to a real-life case study of a mini-model of a NF using Simulink-MATLAB.

## Analysis of measurements for the NRR facility real case study

One of the most essential tasks in nuclear safety is to keep a nuclear facility's electrical system in perfect condition. The safety function is to guarantee that devices and other equipment have enough, high-quality, and suitable power to fulfil safety functions when necessary. Constant and good power quality monitoring helps put our hands on power quality problems, which leads to reduced losses in the production process^10^. So these difficulties lead to higher running expenses and a shorter usable life for the system's equipment. Any disruption causes the reactor to shut down, resulting in a significant financial loss. Power quality monitoring also helps in increasing plant productivity, ensuring efficient equipment performance, determining the need for mitigation equipment, and assessing process equipment sensitivity to disturbances^7^.

### Power quality measurement setup and methodology^11^

**Figure 1:**
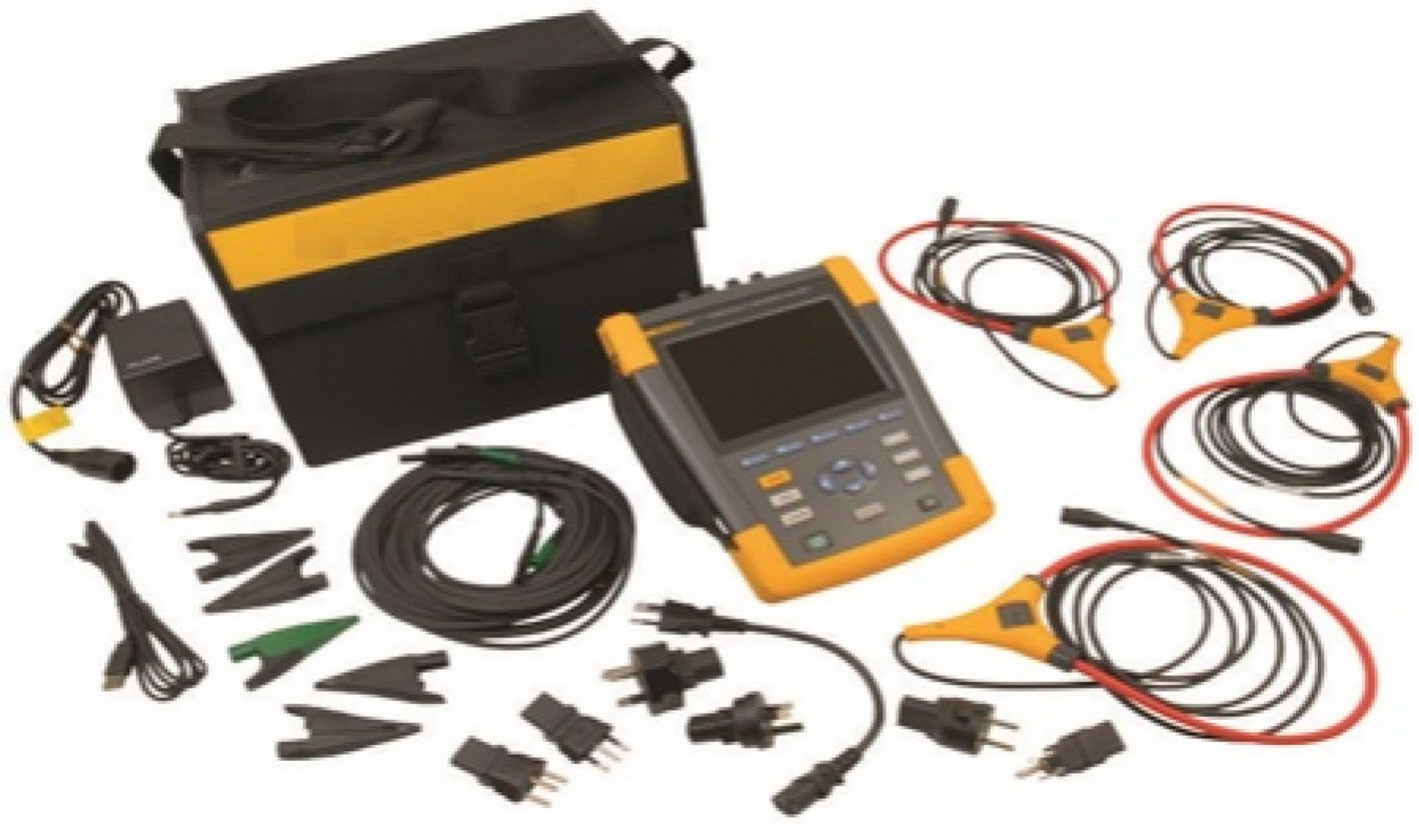
Power quality analyzer device^11^.

It is critical to develop a power quality monitoring system in order to limit the consequences of power quality issues on ageing. In this paper, power quality monitoring and measurements are carried out using Fluke 435-II power quality analyzers. The Fluke device is connected at the 11 kV primary distribution medium voltage terminals in the zone NRRF area to monitor power quality events. The Fluke device consists of a transducer with high bandwidth, a storage unit, an analogue conditioning block, analogue-to- digital converter, and digital signal processing. The power wave function records rapid RMS values with a precision of ± 0.2% of the nominal voltage, allowing investigation of voltage, current, and frequency interactions. According to IEC61000-4-30, the configurable nominal voltage range is 1–1000 V. It has an 8-channel, 16-bit analogue-to-digital converter with a sampling rate of 200 kS/s. True-RMS, peak voltage and current, frequency, swells, transients, interruptions, power consumption, harmonics, inter-harmonics, flicker, mains signals, inrush, and imbalance may all be measured with this device. The instrument triggers and automatically saves the voltage and current waveforms in all three phases and neutral whenever an event or voltage distortion is detected. This method captures hundreds of dips, swells, interruptions, and transients. Voltage transients of up to 6 kV may be recorded for as little as 5 ms. Various electrical parameters were measured in the NRRF electrical system. Figure 1 illustrates the power quality analyzer that was used to perform these observations.

**Figure 2:**
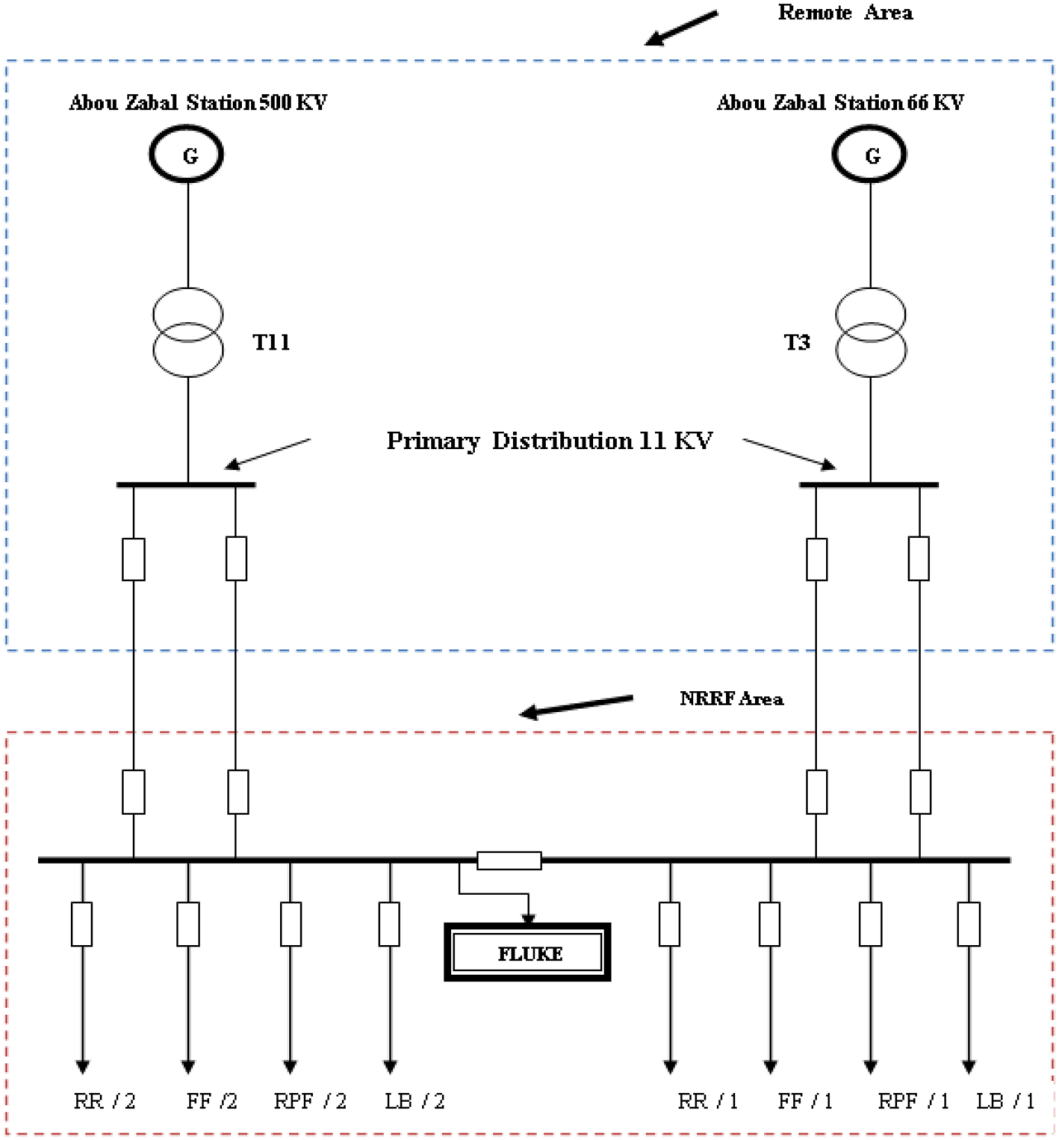
Location of fluke power quality analyzer in NRRF.

The analyzer was installed at the point of common coupling (PCC) on the medium voltage side of the grid in order to measure and evaluate the collective behaviour of the individual nuclear research reactor facility feeders. A substation potential transformer is used to link the Fluke 435 power quality analyzer to the group control breaker (PT). Fluke power quality analyzer measurements are carried out at the NRRF area as shown in Fig. 2.

The power loads of the NFs were being classified as follows:

- Grade ‘A’ loads: As these loads had critical grade of safety, they required the use of Uninterruptible Power System (UPS). With 30-min autonomy, The UPS capability meets all class ‘A’ load demands and conditions.
- Grade ‘B’ loads are those that can be readily reconnected to the system in the event of an interruption in electrical power from the external lines. If the external lines malfunction, the NF has two diesel generators intended to generate enough AC power to power grade ‘B’. Each of these two generators has a capacity of 300 kVA.
- Grade ‘C’ loads: They allow for a brief interruption in power delivery. A conventional power supply was used to power them. Each facility receives power from two separate sources via an 11 kV connection. Transformer T1 is fed by Feeder 1, while Transformer T2 is fed by Feeder 2. The right bus bars are fed by transformer T1, while the left bus bars are fed by transformer T2.

### Case study

**Figure 3:**
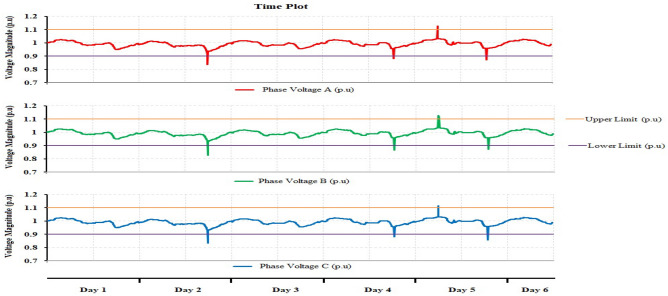
Time plot of phase A, B and C Voltages magnitude.

The measurements are recorded for nearly fourteen days since the nuclear research reactor facility is at maximum power during that period. The nominal voltage is 1 pu. The higher and lower voltage limitations are shown by the purple and orange lines above and below the normal voltage, which have values of 1.1 pu and 0.9 pu, respectively. Sag refers to a reduction in voltage below the lower voltage limit, while swell refers to an increase in voltage over the upper limit. The discussion of the results and analysis is shown as follows:

The time plot of voltage magnitude for the evaluation period is depicted in Fig. 3. During six days of monitoring, the magnitude of the voltage decreased three times below the lower limit and rose once above the higher limit. During the research period, there was no interruption.

**Figure 4:**
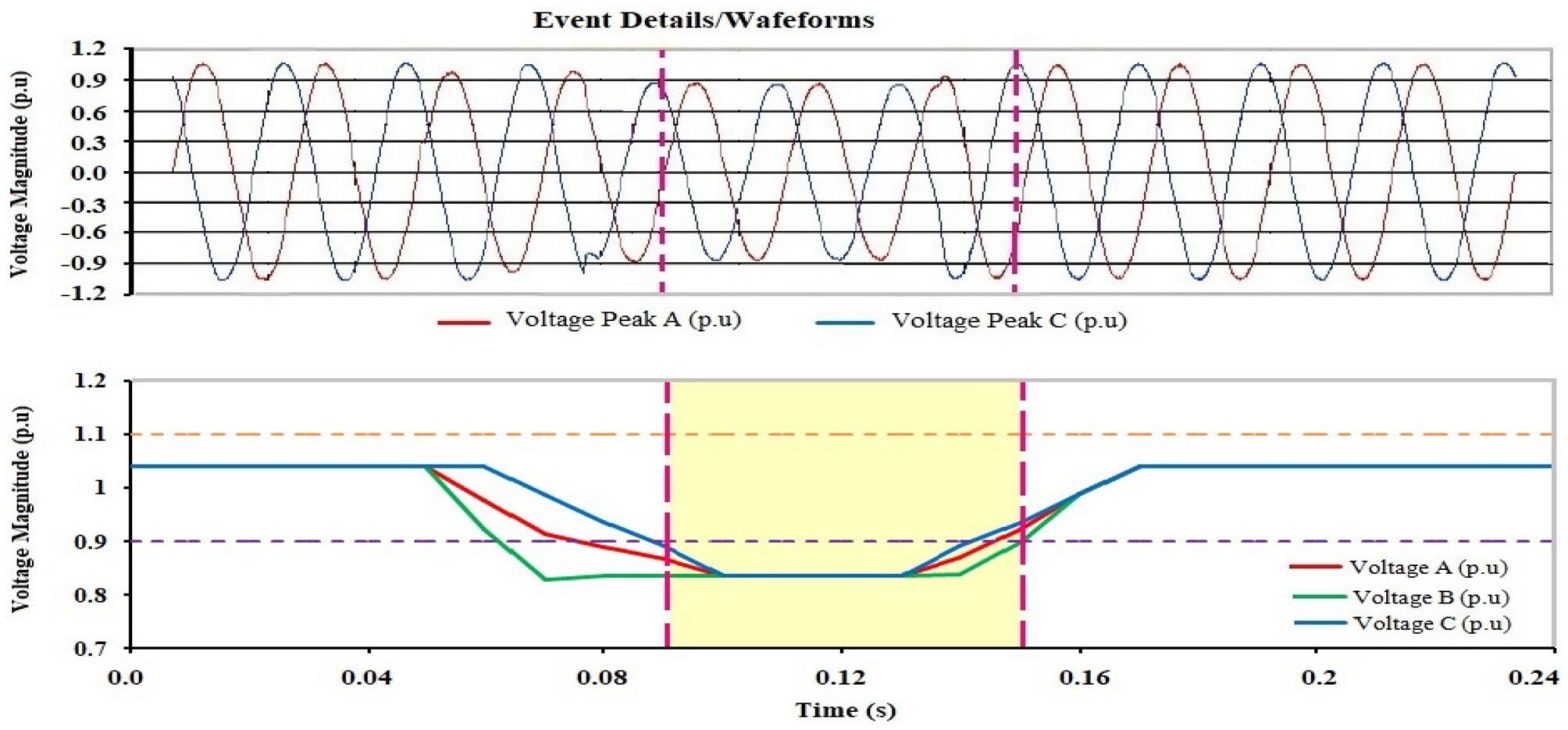
Short duration instantaneous sag.

Referring to the time diagram explained in Fig. 3, it clearly shows that there were three sag voltage events appearing on the second day, the fourth day, and the fifth day, respectively. The values of sag voltage are 0.8364 pu, 0.8819 pu, and 0.8728 pu for days 2, 4, and 5, respectively. One of the three sag events is shown in Fig. 4. The dip in voltage in this event is related to a rapid change in load and the start of the motor. The instantaneous sag period for the duration of 0.06002 s. On the other hand, the other two low voltage values took approximately 0.05503 s and 0.038715 s.

**Figure 5:**
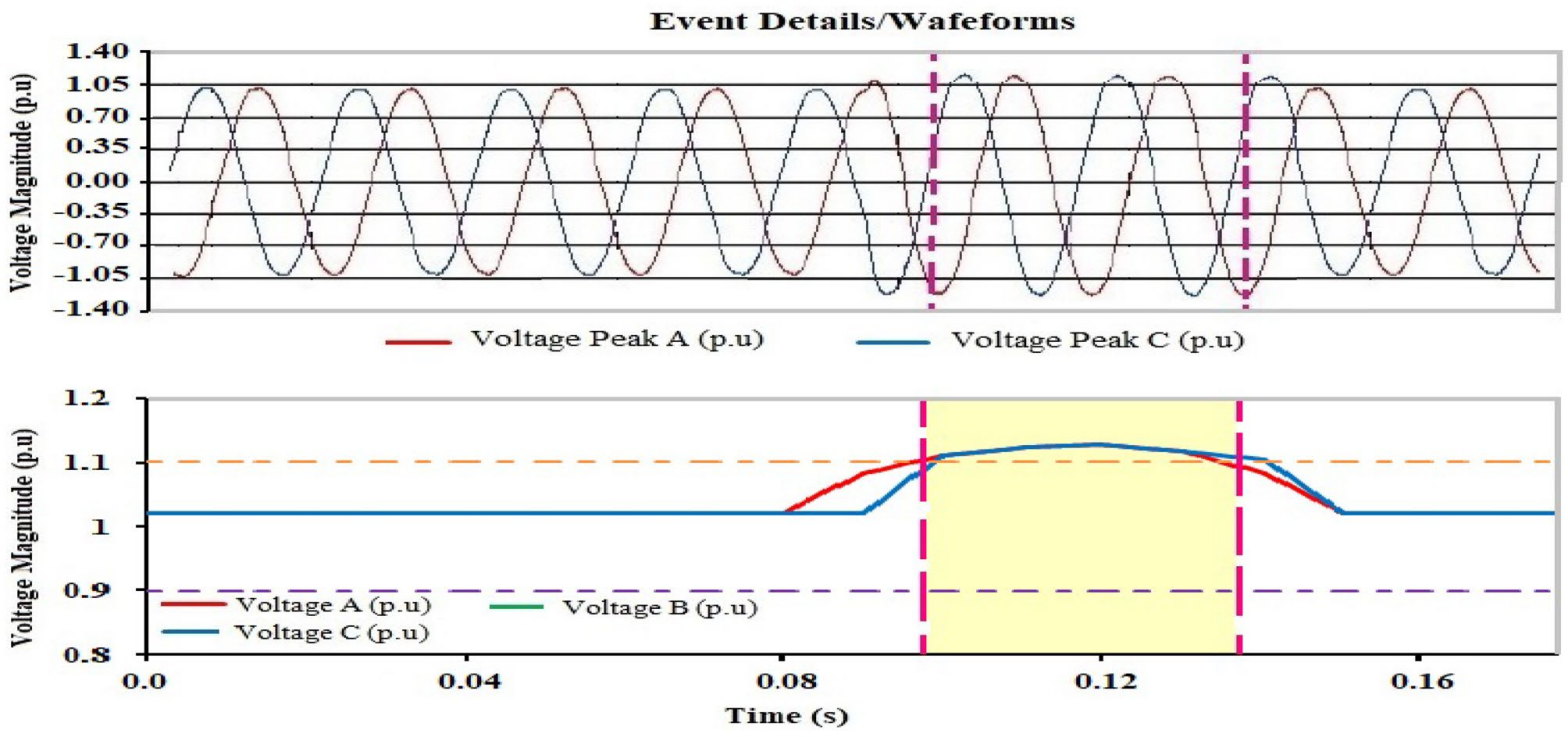
Short duration instantaneous swell for Case 1.

Also, referring to the time plot explained in Fig. 3, only the instantaneous voltage swell event was recorded and appeared on the fifth day. As a result, the voltage rises over its nominal value from 1.0 to 1.1266 pu for the duration of 0.03283 s, as indicated in Fig. 5.

**Figure 6:**
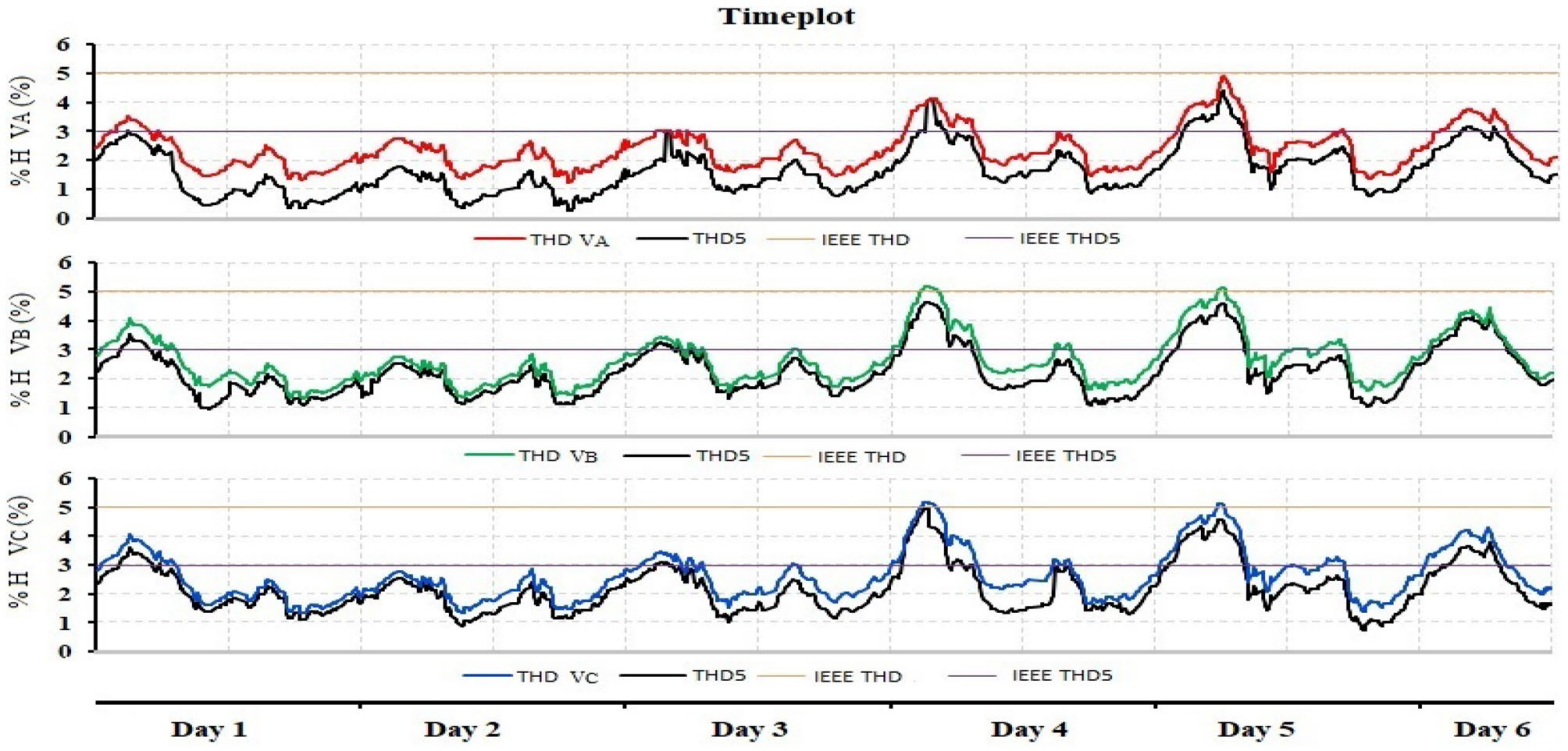
Time plot of THD for phase A, B and C.

Figure 6 explains that no event was recorded for phase A, but two events were recorded for each of phases B and C. The two values of THD for phases B and C are the same: 5.1749% and 5.1301%. These values are very simple, and the rate of change in THD is not large, so they can be neglected.

## The proposed methodology

**Figure 7:**
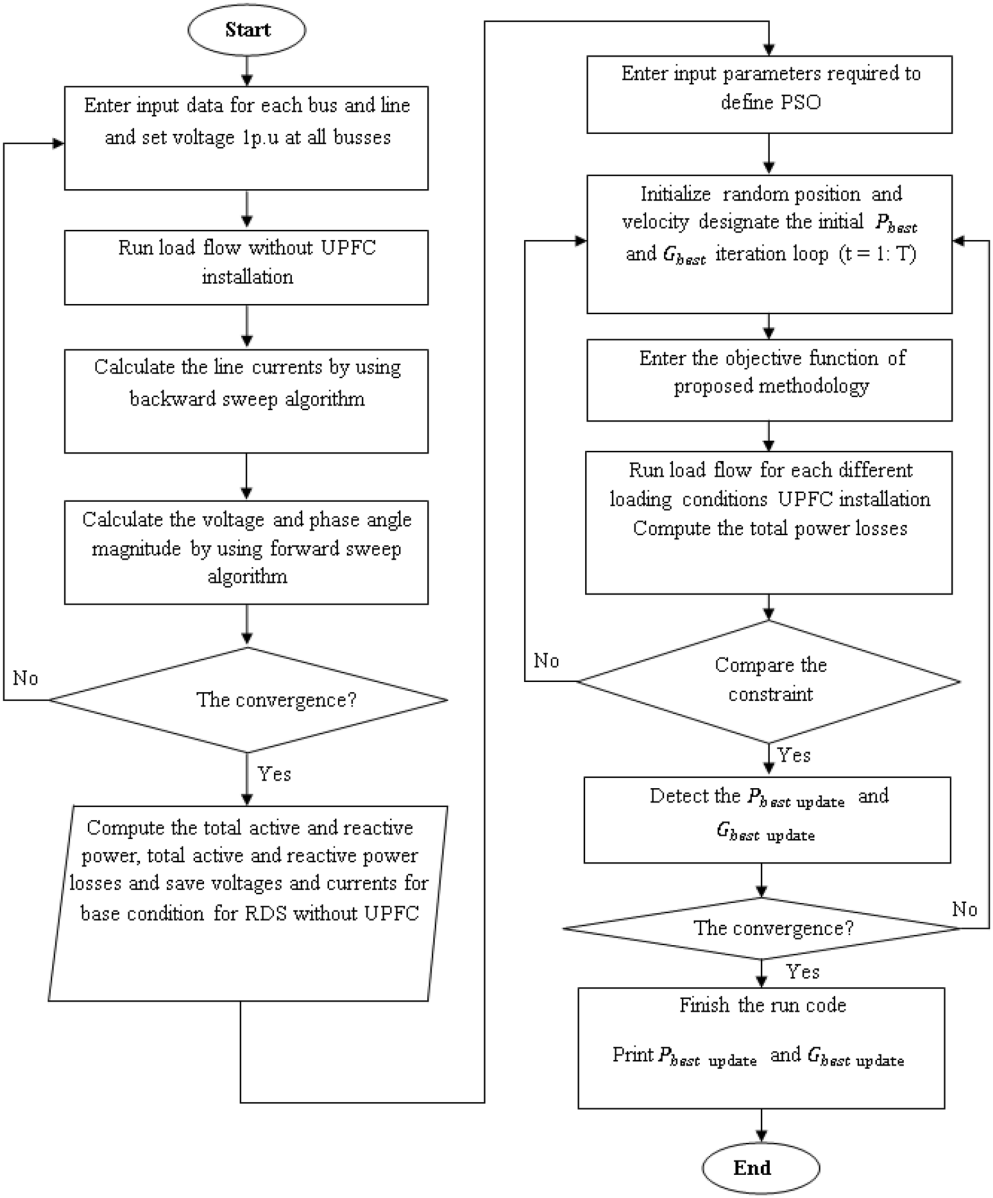
Flow chart depicting the processes for executing the suggested technique.

The suggested technique code for the IEEE standard bus system was first confirmed in our earlier work in Ref.^1^ and then deployed to a tiny model of a NF in this study. The procedure for applying the suggested technique is represented in the flowchart in Fig. 7.

### Power flow analysis in radial distribution system (RDS) ^1^

Reference^1^ explains in detail the RDS's single-line diagram (SLD). As it consists of two buses connected by a line, the SLD represents a simple section of the radial distribution system. Busses y and g are the sending and receiving busses.

### Mathematical model^1^

The mathematical model was discussed in this section; however, it has been thoroughly researched in a reference^1^.

1 $${P}_{yg}={P}_{g}+{P}_{{\text{Loss}}\left(yg\right)}$$

2 $${Q}_{yg}={Q}_{g}+{Q}_{{\text{Loss}}\left(yg\right)}$$

3 $${{\text{I}}}_{{\text{yg}}}=\left(\frac{{{\text{P}}}_{{\text{yg}}}-j{{\text{Q}}}_{{\text{yg}}}}{{{\text{V}}}_{{\text{g}}}\mathrm{\angle }-{\alpha }_{{\text{g}}}}\right)$$

4 $${{\text{I}}}_{{\text{yg}}}=\left(\frac{{{\text{V}}}_{{\text{y}}}\mathrm{\angle }{\alpha }_{{\text{y}}}-{{\text{V}}}_{{\text{g}}}\mathrm{\angle }{\alpha }_{{\text{g}}}}{{R}_{yg}+j{X}_{yg}}\right)$$

From Eqs. (3) and (4), it can be discovered that:

5 $${V}_{y}^{2}-{V}_{y}{V}_{g}\mathrm{\angle }\left({\alpha }_{g}-{\alpha }_{y}\right)=\left({{\text{P}}}_{{\text{yg}}}-j{{\text{Q}}}_{{\text{yg}}}\right)\left({R}_{yg}+j{X}_{yg}\right)$$

By comparing the real and imaginary components of the equations on both sides in (5)

6 $${V}_{y}{V}_{g}*{\text{cos}}\left({\alpha }_{g}-{\alpha }_{y}\right)={V}_{y}^{2}-\left({{\text{P}}}_{{\text{yg}}}{R}_{yg}+{Q}_{yg}{X}_{yg}\right)$$

7 $${V}_{y}{V}_{g}*{\text{sin}}\left({\alpha }_{g}-{\alpha }_{y}\right)={Q}_{yg}{R}_{yg}-{{\text{P}}}_{{\text{yg}}}{X}_{yg}$$

After squaring and adding (6) and (7)

8 $${V}_{g}^{2}={V}_{y}^{2}-2\left({{\text{P}}}_{{\text{yg}}}{R}_{yg}+{Q}_{yg}{X}_{yg}\right)+\left({R}_{yg}^{2}+{X}_{yg}^{2}\right)\left(\frac{{{\text{P}}}_{yg}^{2}+{{\text{Q}}}_{{\text{yg}}}^{2}}{{\left|{{\text{V}}}_{{\text{y}}}\right|}^{2}}\right)$$

9 $${P}_{{\text{Loss}}(yg)}={I}_{yg}^{2}*{R}_{yg}$$

10 $${{\text{Q}}}_{{\text{Loss}}({\text{yg}})}={I}_{yg}^{2}\times {{\text{X}}}_{{\text{yg}}}$$

### Particle swarm optimization (PSO) method^1^

PSO is a swarm intelligence derived from the social behaviour and dynamic movements of insects, birds, and fish. PSO uses particle counts to represent a swarm that moves about the search perimeter in pursuit of the optimal solution. Each particle in the search perimeter modifies its “flying” based on its own and other particles' flying experiences. The PSO algorithm's main goal is to use a velocity vector to update the new position of each particle in the swarm. Using Eqs. (11) and (12) to get the updated location and velocity of each particle

11 $${v}_{i}\left(t\right)=\Theta\times {v}_{i}(t-1)+{c}_{1}*{r}_{1}\left({P}_{best, i}-{x}_{i }\left(t-1\right)\right)+{c}_{2}*{r}_{2}\left({G}_{best, i}-{x}_{i }\left(t-1\right)\right)$$

12 $${{\text{x}}}_{\mathrm{i }}\left({\text{t}}\right)=\left({{\text{x}}}_{\mathrm{i }}\left({\text{t}}-1\right)+{{\text{v}}}_{{\text{i}}}\left({\text{t}}\right)\right)$$

### The objective function

The suggested technique employs total power loss as an objective function that should be reduced within certain limits. The suggested approach for optimizing UPFC device characteristics and its best placement formulation is implemented in MATLAB and tested on a typical IEEE-33 bus radial distribution system. The suggested methodology's objective function, which is used to decrease total power loss under specified limitations given in Eqs. (13) and (14).

13 $$Objective\,function= Min. \,S_{Total\,Loss} ={\left[{\left(\sum_{y=1}^{Nl} {P}_{Loss\left(yg\right)}\right)}^{2}+{\sum_{y=1}^{Nl} {Q}_{Loss(yg)}}^{2}\right]}^{1/2}$$

14 $$Inequality\,constraints= \begin{array}{c}{P}_{total\,load}^{min}\le {P}_{upfcy,\,size}\le {P}_{total\,load}^{max}\\ {Q}_{total\,load}^{min}\le {Q}_{upfcy,\,size}\le {Q}_{total\,load}^{max}\\ \left|{V}_{i}^{min}\right|\le \left|{V}_{i}\right|\le \left|{V}_{i}^{max}\right|\\ 0.9p.u\le {V}_{bus}\le 1.1p.u\\ 0\le {\theta }_{s}\le 2\pi \\ 0\le {I}_{sh}\le {I}_{sh}^{max}\end{array}$$

### Comparison of the proposed PSO with other methods on 33-bus RDS^1^

On the IEEE 33-bus RDS, the capability of the installation of the UPFC device at line 5 is evaluated. The suggested PSO method is utilized to determine the best allocation for the UPFC device in order to minimize overall power loss in relation to the voltage profile within the range. The simulation results for this test system with the UPFC device installed are summarized in Table 1 under the proposed PSO designation and compared to the other works. Table 1 shows that the suggested PSO has the lowest total power loss of all the algorithms provided. The suggested PSO approach achieves a substantially lower minimum voltage after optimization than some of the methods shown in this table, and the system's voltage profile is also enhanced. The CPU time for each method is shown in Table 1. Although SPSO^12^ and SPSO-SBAT^13^ have faster convergence than the proposed PSO, the CPU time for the proposed PSO is faster than the other approaches listed in Table 1. As a result, it should be highlighted that the suggested PSO algorithm outperforms existing algorithms in terms of loss reduction and convergence characteristics.

**Table 1:** Comparison of the proposed PSO with other methods on 33-bus RDS.

Approach	Equipment/tie switch	Location	Power losses (kW)	Loss reduction (kW)	Minimum voltage after optimization (pu)	Enhancement of voltage (pu)	Run time (s)
Proposed PSO	UPFC	**5**	**111.2759**	**90.7413**	0.9398	**0.0449**	5.85
SPSO^12^	33,34,35,36,37	–	112.58	89.0143	0.9500	0.0366	**4.02**
Firefly^13^	7,9,14,32,37	–	142.8	58.7943	0.93782	0.02442	8.121
SPSO^13^	7,9,14,32,37	–	140.42	61.1743	NM*	NM*	**6.173**
SBAT^13^	7,9,14,32,37	–	141.20	60.3943	NM*	NM*	**6.025**
BA^14^	DSTATCOM	30	143.38	58.2143	0.9260	0.0126	6.5
BA^14^	DSTATCOM	11,24,30	132.08	69.5143	0.9361	0.0227	9.62
PSO^15^	DG	30	151.38	50.2143	0.9500	0.0366	NM*
EPSO^16^	11,28,32,34	–	120.7	80.8943	**0.9980**	0.006	12.2
EP^16^	17,7,1037,13	–	125.2	76.3943	**0.9980**	0.006	55
PSO^16^	7,10,28,14,32	–	126.4	75.1943	0.9975	0.0055	16.0
IA^17^	DSTATCOM	12	171.81	29.7843	0.9258	0.0124	21.22
TLBO^18^	DG	14,29,30	126.496	75.0983	0.9302	0.0168	12.64
QOTLBO^18^	DG	14,27,33	115.425	86.1693	0.9324	0.019	12.58
GA^19^	7,9,14,32,37	–	139.5101	62.0842	0.9297	0.0163	NM*
AC^19^	7,9,14,28,32	–	139.9383	61.6560	0.92977	0.01637	NM*
IAICA^19^	7,9,14,32,37	–	139.5101	62.0842	0.9500	0.0366	NM*
FWA^20^	7,14,9,32,28	–	139.98	61.6143	0.9413	0.0279	6.4
HAS^21^	7,10,14,37,36	–	138.067	635,273	0.9342	0.0208	7.2
RGA^22^	7,9,14,32,33	–	139.532	62.0623	0.9315	0.0181	13.8

NM*: not mentioned in the given reference, and the best findings are bold.

## Description of single line diagram and Simulink model for NRR (real case)

**Figure 8:**
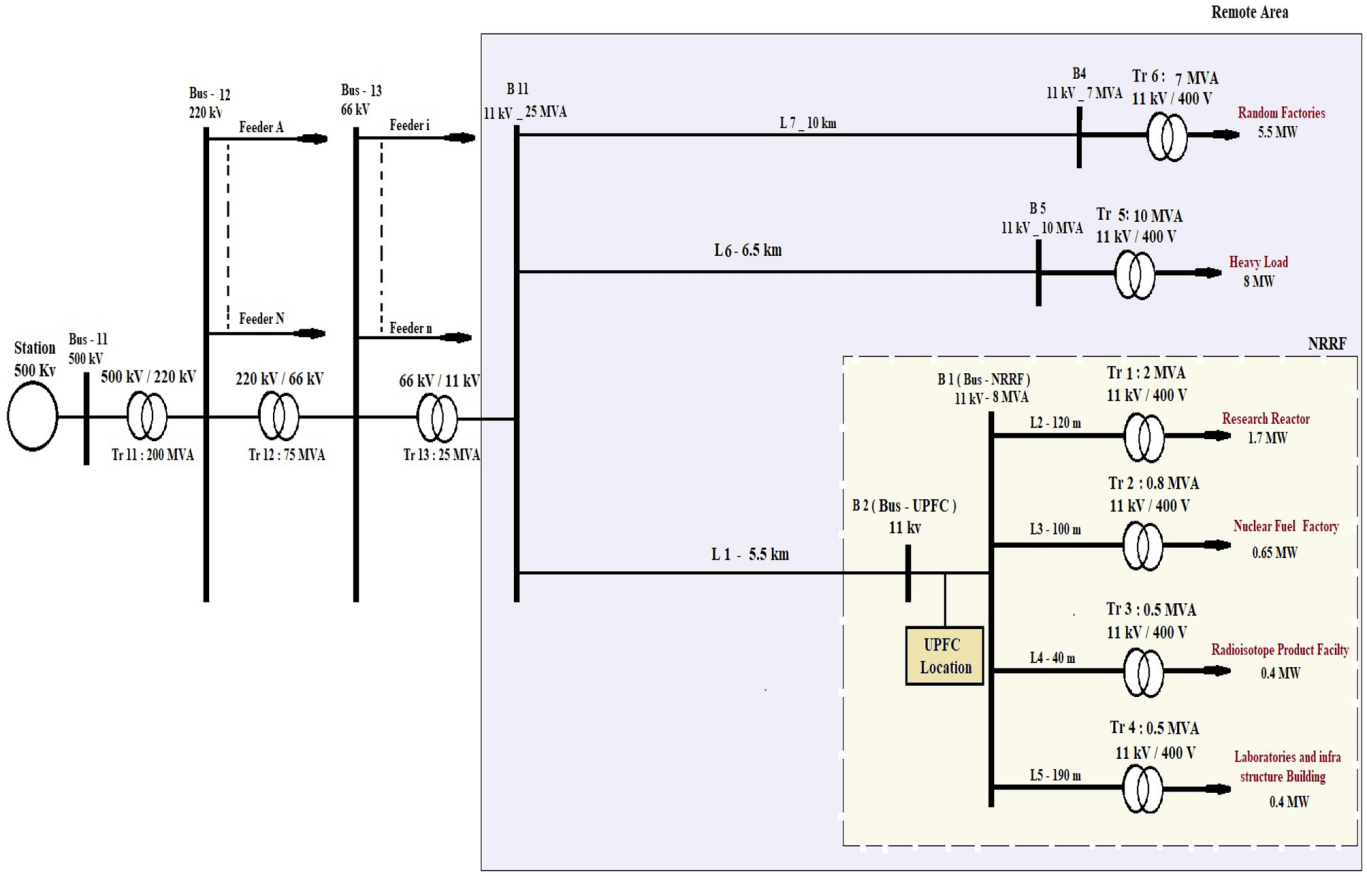
Single line diagram of UPFC based nuclear research reactor facilities.

**Figure 9:**
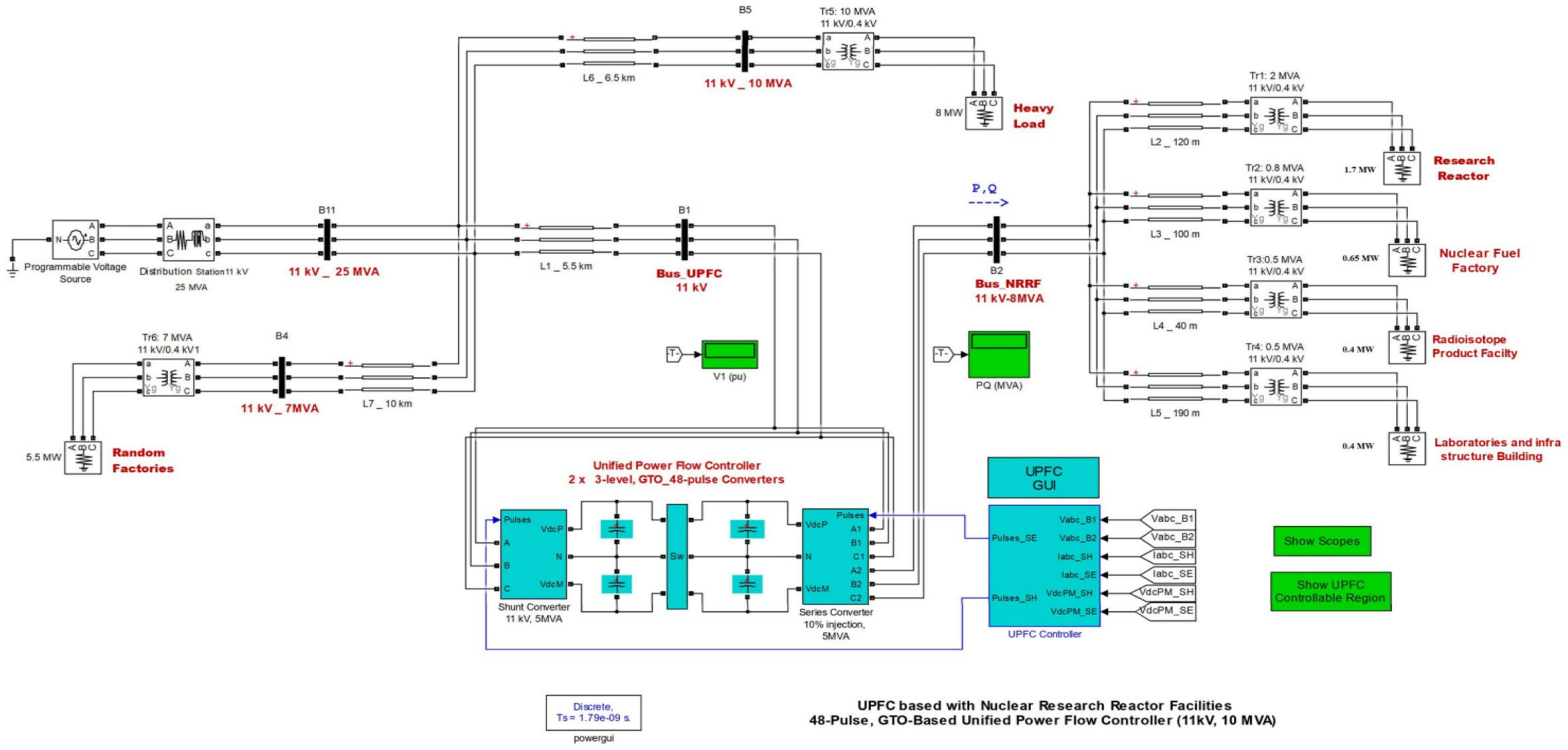
MATLAB Simulink model of UPFC based nuclear research reactor facility.

The single-line diagram and simulation model for UPFC based on NRRF are shown in Figs. 8 and 9, respectively. The Simulink Model, shown in Fig. 9, represents the UPFC based on the real case for the NRRF, which consists of four facilities: the RR, FF, RPF, and LB, which are 1.7 MW, 0.65 MW, 0.4 MW, and 0.4 MW, respectively. The system is connected in a loop and consists of five buses, seven transmission lines, and six 11 kV/0.4 kV transformer banks. The UPFC, installed at the right end of the 5.5-km line L1, between the 11 kV buses Bus_UPFC and Bus_NRRF, is used to regulate the active and reactive power flowing through Bus_NRRF while keeping control voltage at Bus_UPFC. It is composed of two 5-MVA, three-level, 48-pulse GTO-based converters, one connected in shunt at bus B1 and one linked in series between Bus_UPFC and Bus_NRRF. A DC bus can be used to exchange power between shunt and series converters.

As the UPFC is an expensive device. So, it is obvious to use it as much as possible. In this matter, the control system used in the UPFC is divided into two types: regular control and modified control. The modified control represents the main contribution of this paper. It simply uses the same controls as regular control but with modifications to the voltage regulator, which will be discussed in detail in the following section. The advantage of using this type of control loop is that it modifies the performance of the active and reactive power flow controls, widens their control range, and enhances voltage sag and swell.

**Figure 10:**
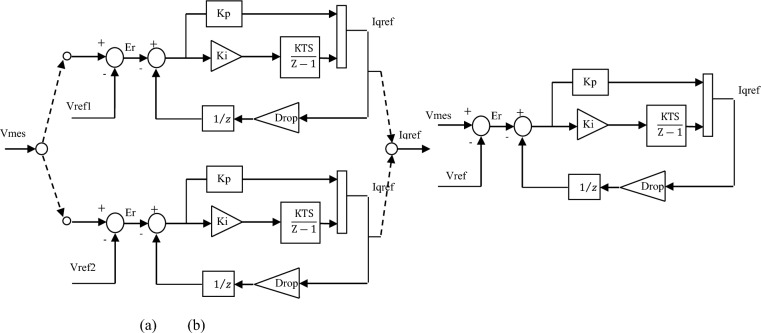
Schematic diagram of (**a**) Modified voltage block, (**b**) Regular voltage.

**Figure 11:**
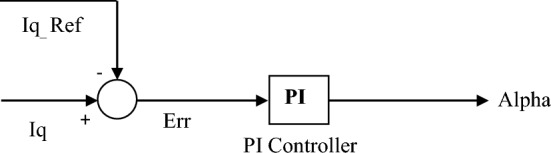
Schematic diagram of current regulator block.

The modified voltage is performed by three PI regulators: the measured voltage *V*_*meas*_ and two reference voltages *V*_*ref*_ (V_ref1_ = 0.9 pu and V_ref2_ = 1.1pu, while V_ref_ = 1pu in regular voltage regulation). The modified voltage block (outer loop) is shown in Fig. 10a, and the regular voltage regulation is shown in Fig. 10b. The outer loop computes the reactive current reference I_qref_ used by the current regulator block (inner loop) shown in Fig. 11, whose output is the α angle.

### Impact of the UPFC on harmonic distortion

The harmonics 5 + 12n (5, 17, 29, 41, …) and 7 + 12n (7, 19, 31, 43, …) are cancelled by the 30-degree phase shift between the Y and secondary. likewise, harmonics 11 + 24n (11, 35, …) and 13 + 24n (13, 37, …) can be deleted due to the 15-degree phase shift between the two transformer groups (1Y and 1 by 7.5°, 2Y and 2D lagging by + 7.5°). Given that the Y and secondary do not transmit all 3n harmonics, the first harmonics that are not cancelled by the transformers are the 23rd, 25th, 47th, and 49th. We must choose the appropriate conduction (sigma) angle to find the optimizing conduction angle (sigma = 0°–180°). Thus, the 47th and 49th harmonics are the first important harmonics. This inverter produces a nearly sinusoidal waveform with 48 steps. This is an essential step; this step is done through simulations by varying the conduction (sigma) angle (σ) from 10° to 180°, and the result is shown in Table 2. This table contains the sigma angle, total harmonic distortion (THD%) before and after the converter, and relative fundamental.

**Table 2:** The result for run simulation.

Sigma (°)	V_an_ and V_ab_ (THD%)	V_abc_ (THD %)	Relative fundamental
10	283.39	73.94	0.0848
60	80.31	7.58	0.5015
110	35.15	6.01	0.8217
150	31.93	7.56	0.969
160	36.16	4.53	0.9879
170	41.76	4.35	0.9993
170.5	42.17	4.17	0.997
171	42.36	4.01	1
**171.5**	**42.63**	**3.89**	**1**
172	42.97	3.82	1.001
172.5	43.28	3.79	1.001

Significant values are in bold.

The best point of operation occurs when, based on Ref.^23^,

- Relative fundamental is 1pu or almost 1pu.
- The frequency must remain between (49.5–50.5 Hz) or $\pm 1\mathrm{\%}$ the fundamental frequency.
- The total harmonic distortion of the voltage must be THD% < 8.

**Figure 12:**
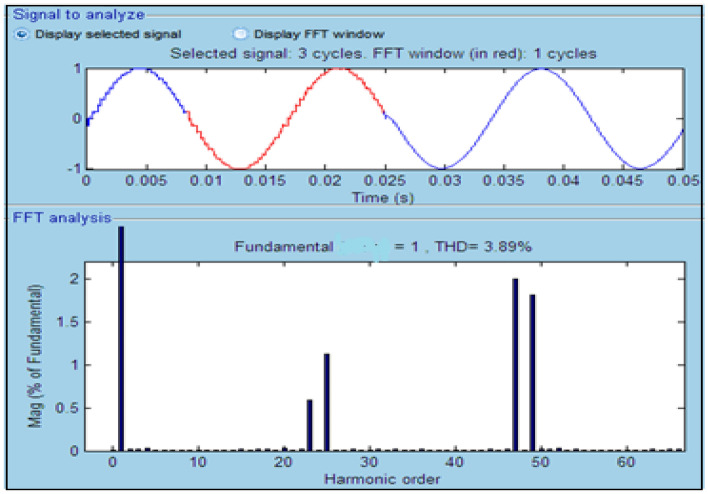
48-pulse inverter output voltage and its harmonic order.

Based on this rule and Table 2, a conduction angle of 171.5° is chosen as an optimal point. The 48-pulse inverter output voltage and associated harmonic order are depicted in Fig. 12. As shown in Fig. 12, the 48-pulse inverter output voltage becomes smooth because most harmonics are filtered.

### Impact of PI controller based UPFC on enhancement of active and reactive power flow control

To specify the active and reactive power flow reference (Case 0), the UPFC is disconnected (without control). The results display that the normal active power flow is P =  + 3.15 MW. So that the reference active power is specified in the simulation at P_ref_ =  + 3.15 pu/ on a 1 MVA base (+ 3.15 MW). Also, the results illustrate that the normal reactive power flow is Q = 2.1254 MVAr. So that, the reference reactive power is specified in Simulink at Q_ref_ = 2.1254 pu/1 MVAr (2.1254 MVAr). The controllable region is obtained by keeping the injected voltage at a maximum value of 10% (0.1 pu) of the nominal line-to-ground voltage (6.351 kV) and varying its phase angle from 10° to 360°.

**Figure 13:**
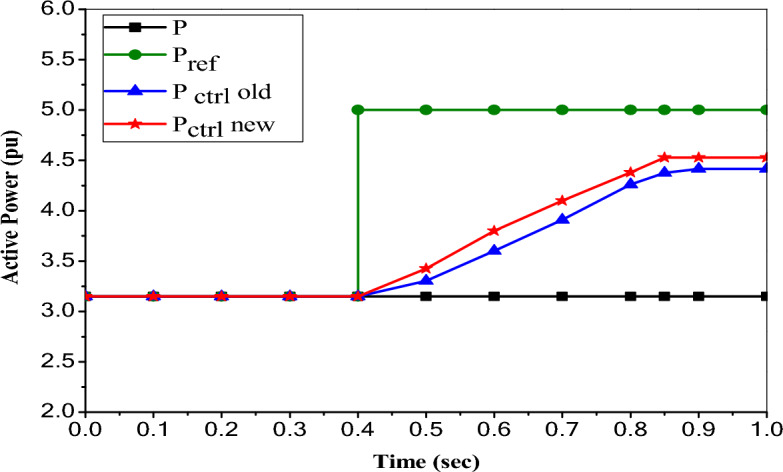
Reference and controlled active power with PI based UPFC.

According to Fig. 13, the controlled active power does not reach the new reference power of 5pu, but instead reaches 4.5267 pu only at ∆t = 0.4497 s and remains constant by enabling the UPFC device with the proposed control. This indicates that the UPFC has a maximum ultimate active power value of 4.5267 pu. When this upper limit is compared by (4.415 pu), the upper limit that occurred with the old control, the upper limit increases by 2.53% of the proposed control loop. So, the injected voltage is increased from 0.0126 to 0.1 pu (V_inj_ = 6.351 kV) throughout the time 0.4497 s, and this value is the maximum value of the injected voltage that can be taken by UPFC. So, the value of active power (P = 4.5267 MW) is the maximum value that this UPFC can handle.

**Figure 14:**
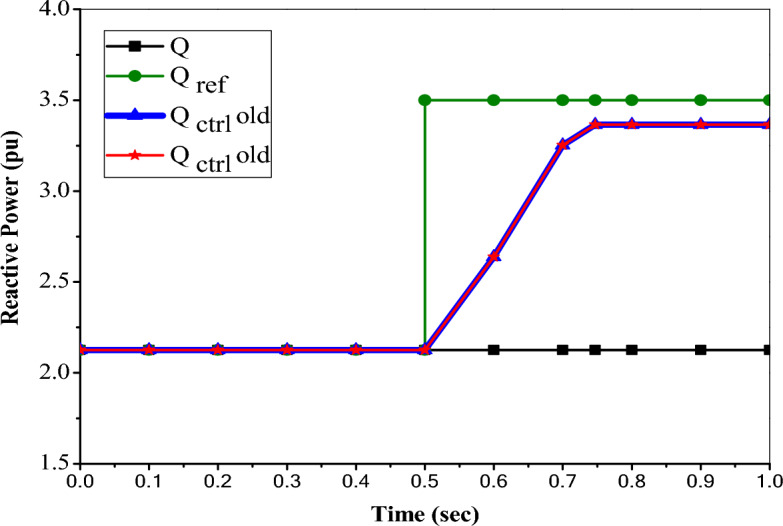
Reference and controlled reactive power with PI based UPFC.

Figure 14 submits that the controlled reactive power reaches only 3.365 pu at ∆t = 0.247 s and remains constant by enabling the UPFC device with the proposed control. So, the upper limit of the reactive power flow described by UPFC is 3.365 pu, which is the same value that occurred with the old control. In this case, the injected voltage is increased from 0.0126 to 0.1 pu (V_inj_ = 6.351 kV) during time ∆t = 0.247 s, and this value is the maximum value of the injected voltage that can be taken by UPFC. So, the value of reactive power (Q = 3.365 MVAr) is the maximum value that this UPFC can handle.

### Impact of the UPFC on voltage sag and swell

The UPFC is used to control either voltage sag only, voltage swell only, or both. The following tests are divided as follows:

- Voltage sag control.
- Voltage swell control.

#### Voltage sag control using UPFC

**Figure 15:**
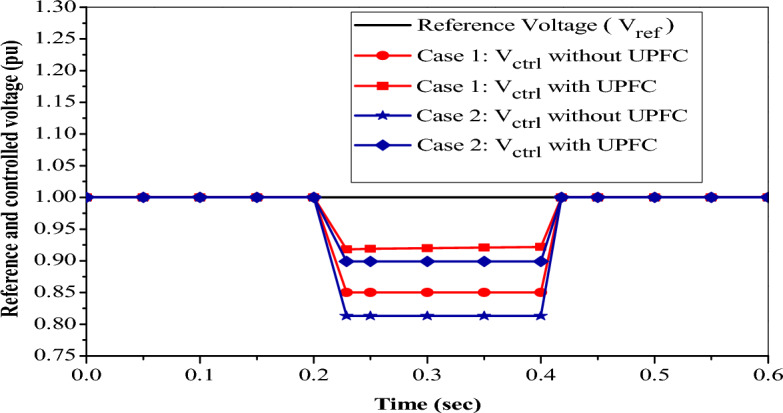
Reference and controlled sag voltage with PI based UPFC.

To demonstrate the control action of the UPFC on the voltage sag, there are two cases to identify the minimum voltage sag occurring, and the UPFC can overcome this problem and inject voltage to an equal reference voltage of 1 pu or standard by generating reactive power to keep voltage at nearly 1 pu. Figure 15 illustrates the impact of applying the UPFC device on NRR during the occurrence of different sag voltages as follows:

In case 1, at a time between t = 0.2 s and t = 0.4 s, the measured voltage V_ctrl_ = 0.85 pu by injecting sag voltage without using UPFC. When the UPFC is used and installed in the system, the measured voltage increases to V_ctrl_ = 0.918 pu which is within the acceptable limit standard^23^, and after t = 0.4 s, the UPFC operating point comes back to its initial value.

In case 2, at a time between t = 0.2 s and t = 0.4 s the measured voltage V_ctrl_ = 0.813 pu by injected sag voltage without using UPFC. When the UPFC is used and installed in the system, the measured voltage increases to V_ctrl_ = 0. 899 pu, but this value is out of the acceptable voltage standard^23^. So, the UPFC improved the voltage value but couldn’t make it to the standard. After t = 0.4 s, the UPFC operating point returns to initially floating.

In this case, the UPFC acts as a capacitive source to overcome the decrease in voltage. So, the minimum value of the sudden voltage decrease is 0.82 pu, at which point the PI-based UPFC can turn the voltage to an acceptable value.

#### Voltage swell control using UPFC

**Figure 16:**
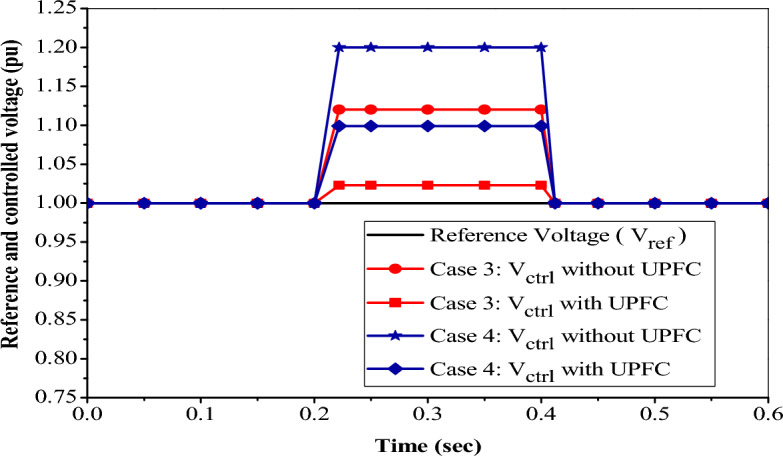
Reference and controlled swell voltage with PI based UPFC.

To demonstrate the control action of the UPFC on the voltage swell, there are two cases to identify the maximum swell voltage, and the UPFC can overcome this problem and inject voltage to an equal reference voltage of 1pu or standard by absorbing reactive power to keep voltage at nearly 1 pu. Figure 16 clarifies the effect of installing the UPFC device on NRR in the event of different swell voltages as follows:

In case 3, at a time between t = 0.2 s and t = 0.4 s, the measured voltage V_ctrl_ = 1.12 pu by injecting swell voltage without using UPFC. When the UPFC is used and installed in the system, the measured voltage decreases to V_ctrl_ = 1. 023 pu, which is within the acceptable limit^23^, and after t = 0.4 s, the UPFC operating point comes back to its initial value.

In case 4, at a time between t = 0.2 s and t = 0.4 s, the measured voltage V_ctrl_ = 1.2 pu without using UPFC. When the UPFC is used and installed in the system, the measured voltage decreases to V_ctrl_ = 1.099 pu, which is a value within the standard limit^23^. After t = 0.4 s the UPFC operating point comes back to initially floating.

In this case, the UPFC acts as an inductive source to overcome the increase in voltage. So, the maximum value of the sudden voltage increase is 1.2 pu, at which point the PI-based UPFC can turn the voltage to an acceptable value.

## Conclusion

In this work, the power quality issues of the electrical system at NF were studied to assess the current state from a power quality standpoint. This is part of a research study aimed at enhancing the performance and efficiency of the electrical system of a nuclear reactor to reach a safe operating condition. The NF is fed from two feeders of different capacities and connected at 11 kV. The first feeder is connected to the 500 kV station, and the other is connected to the 66 kV station. The results of the comparison between them showed that the 500 kV plant is better and more appropriate than the 66 kV plant, given the time of operation of the research reactor. Therefore, a 500 kV plant is recommended when the nuclear research reactor is in actual operation. However, the results show some power quality issues that still hamper safe operation (for example, enhance power flow, improve voltage sag and swell, and improve transient stability). The following study will provide some solutions to overcome it.

In this paper, a proposed approach is provided to reach the safe limit of the dependability of the electrical system at a Nuclear Research Reactor (NRR) facility. This paper presents a novel approach to employing an UPFC device to manage both active and reactive power flow, mitigate voltage sag and swell, minimise total Power Loss (PL) and harmonics while keeping the voltage within a specific limit. It also presents different UPFC controllers, i.e., PI-based UPFC. The simulation results make it clear that the new PI controller-based UPFC for the described UPFC stretched the maximum final active power value by 2.53% from the old PI controller and improved voltage sag , swell up to 18% and 20%, respectively, for the prescribed UPFC while keeping the output in the standard range. Finally, the results show that the proposed technique is a good method to improve feeding ageing for NRR (Supplementary Information).

### Supplementary Information

Supplementary Information.

## Data Availability

All data generated or analysed during this study are included in this published article.

## Abbreviations

FACTS: Flexible AC transmission systems
UPFC: Unified power flow controller
VSC: Voltage source controller
NRRF: Nuclear research reactor facility
RR: Research reactor
FF: Fuel factory
RPF: Radioisotope product facility
LB: Laboratories and infra-structure building
RDS: Radial distribution system
STATCOM: Static synchronous compensator
SSSC: Static synchronous series compensator
PQ: Power quality
PWM: Pulse width modulation
SBAT: Self-bat algorithm
TLBO: Teaching learning-based optimization
QOTLBO: Quasi-oppositional teaching learning based optimization
FWA: Fireworks algorithm
THD: Total Harmonic Distortion
${{\text{P}}}_{{\text{g}}}$: Total effective active power
${{\text{Q}}}_{{\text{g}}}$: Total effective reactive power
${{\text{P}}}_{{\text{Loss}}\left({\text{yg}}\right)}$: Active power losses
${{\text{Q}}}_{{\text{Loss}}\left({\text{yg}}\right)}$: Reactive power losses
${{\text{I}}}_{{\text{yg}}}$: Current flow between buses y and g
${\mathrm{\alpha }}_{{\text{y}}}$: Voltage angles buses y
${\mathrm{\alpha }}_{{\text{g}}}$: Voltage angles buses g
