# Supplementary material for: Analytical study and real simulation for improving the safety of ageing nuclear facility using UPFC
Source: Sci Rep. 2024 Jan 2;14:210. doi: 10.1038/s41598-023-50356-1 (PMC10762254; doi:10.1038/s41598-023-50356-1)
Supplement: Supplementary file 1 — Supplementary Information. [file 41598_2023_50356_MOESM1_ESM.docx]

**FLUKE 435 power quality analyzer**

In order to measure and analyze power quality problems in grid-connected NRRF network, fluke 435 power quality analyzer is installed in the group control breaker of 11 kV bus bar. Various power quality events such as sag, swell, transient, interruption, harmonics, and flicker are found during the measurement period. Measurements are recorded for approximately 6 days per feeder and the nuclear research reactor facility is at maximum power during that period.

**Scenario A:-**

The device was connected to a 500 kV station for 6 continuous days and the actual operating time. Over a period of 6 days, the readings were taken daily and collected in an Excel sheet for analysis and compiled together in one week for the drawing in Excel ending with Feeder 1. In the beginning, the readings were taken as V1 RMS (KV)) and then they were converted to V1 RMS (PU))) as It is shown in column G so that we can draw it, and the Upper and Lower limits were determined as shown in columns I and J. Then this relationship between time and effort was drawn for a period of 6 days. Likewise, work was done on the second and third efforts, then they were all collected in one drawing.

**Scenario B:-**

In this scenario, the device was connected to a 66 kV station for a period of 6 continuous days during the actual operation of the station. Over a period of 6 days, the readings were taken daily and collected in an Excel sheet for analysis and compiled together in one week for the drawing in Excel ending with Feeder 2.

**M-file - IEEE -33 RDS and UPFC**

After taking measurements and analyzing them on the nuclear reactor network, we concluded that there are some problems in power quality that negatively affect the safe operation of a nuclear facility. In this chapter, these issues will be addressed using a UPFC device, and the optimal location will be chosen using an artificial intelligence method called PSO. This method will initially be applied to the IEEE -33 RDS bus to ensure the quality and effectiveness of the method used.

The program for the results of the sixth chapter consists of a file (m-file) to define the parameters of the studied radial distribution system (IEEE -33 RDS), and **the results were plotted using the Origin pro 8 program. In this thesis, the load flow analysis was solved using the forward/backward scanning algorithm.** This method allows us to know the results of data in each bus and line such as voltage, active and reactive power flow, and active and reactive power losses. In this chapter, initially the basic case load flow analysis was studied without specifying the location of the UPFC and the voltage of each bus, and the active and reactive power losses of each line in the radial distribution under different load conditions were calculated. During the study, three different loads were considered which will be named as follows: low load (SL = 60%), normal load (SN = 100%) and high load (SH = 120%(.

An RDS analysis was scheduled for the three different loading conditions before using the UPFC device using the PSO algorithm of the optimization approach

**Results and discussion of optimal UPFC device allocation in an IEEE – 33 bus test systemwhich were published in a previous paper under the name " *A sustainable solution to ensure the dependently and safety of electrical grid relying on optimal allocation of UPFC for research reactor"* Kerntechnik 2022**.

**Part of this paper**

**The model presented in section 4.1** is used for an IEEE – 33 bus radial distribution system, with the objective function of minimizing the total apparent power loss, which must be less than the base case loss of 65.1097 KVA, 242.3691 KVA and 342.6763 KVA for low load, normal load and high load, respectively. The objective function must be gotten under the constraint **that discussed in section 3.4**. To find the optimal allocation for UPFC device, we put it at each line and calculate the total apparent power losses and compare the results with the base case losses. Figure 7 shows the apparent power loss with various placement of UPFC device in the IEEE-33 bus system. refer to this figure, we found the lower values for the total apparent power losses at line 5 (between bus 5 and bus 6). The total apparent power losses at line 5 at low load condition, normal load condition and high load condition are 50.8742 KVA, 155.5721 KVA and 202.3065 KVA, respectively. So, the line 5 is the most suitable placement for putting the UPFC device for minimizing losses and minimizing voltage deviation. The UPFC parameters that’s achieved the objective function are *V_s_= 0.067,Ɵ_s_ = 0.055 and I_sh =_ 0.022*. Since, there is a wide range of loss values, so we taking into account the logarithm scale on the Y axis to get a reasonable vision. **The suggested approach was coded in MATLAB and run on an Intel Core i5 PC with a 2.6 GHz processor and 4 GB of RAM.**

**This study ignores the number of UPFC devices. On the other wise, it takes in to account the best results obtained by only one UPFC device with the minimum cost.**

Figure 7. Total apparent power loss at different location of UPFC device in IEEE – 33 bus system

**5. 1 Low Load Condition after optimization**

The active power losses of each line without and with UPFC device on line 5 allocation are illustrated in Figure 8.As seen in Figure 8, line 2 contributes the most to the maximum active power loss of the system without UPFC device allocation, followed by line 5, line 3, and line 4. With UPFC device allocation at line 5, there is obvious reduction in active power loss at each lines. The active power loss at line 2 reduces to 9.7917 kW and other lines 3, 4, and 5 have active power loss less than the base case at low load i.e. without UPFC device allocation.

Figure 8. Active power losses in each line of IEEE - 33 bus system without and with UPFC allocation at low load

Figure 9presentedthereactive power losses of each line without and with UPFC device on line 5 allocation. It clearly shows, line 5 contributes the maximum reactive power loss of the system without UPFC device allocation, followed by line 2, line 1, and line 27. With UPFC device allocation at line 5, there is obvious reduction in reactive power loss at each lines. The reactive power loss at line 5 reduces to 2.8857 kVAR. The reactive power loss at lines 1, 2, and 27 when using UPFC device allocation are1.885 kVAR, 3.7512 kVAR and 1.8833 kVAR, respectively. This results have less than the base case at low load i.e. without UPFC device allocation.

Figure 9. Reactive power losses in each line of IEEE - 33 bus system without and with UPFC allocationat low load

When UPFC device is allocated at line 5 then the total active, reactive and apparent power losses at low load are 42.6926 kW, 27.6682 kVAR and 50.8742 KVA, respectively. The related losses are minimized after optimization by 22.16 %, 21.14 % and 21.86%, respectively shown in Figure 10.

Figure 10. Total active, reactive and apparent power losses of IEEE 33 bus system without and with UPFC device allocationat low load

The voltage for each bus at low load condition of the IEEE – 33 bus system without and with UPFC device located at line 5 is presented in Figure 11. In spite of the voltages on all buses in this condition without UPFC device are within the voltage regulation range but when allocated UPFC in line 5, The voltages have been improved. The minimum voltage level without and with UPFC device allocated at line 5 of the system are 0.9577 p.u. and 0.9719 p.u., respectively.

Figure 11. voltages profile of IEEE - 33 bus system without and with UPFC device allocation at low load

**5. 2 Normal Load Condition after optimization**

Figure 12 indicates that the active power losses of each line without and with a UPFC device on line 5 allocation. Line 2 contributes the most to the system's maximum active power loss without UPFC device allocation, as shown in Figure 12, followed by line 5, line 3, and line 4. There is a drastic reduction in active power loss at each line when using the UPFC device allocation at line 5. The active power loss at normal load at line 2 has been reduced from 51.5711 kW to 22.7833 kW after putting the UPFC device allocated in line 5. Other lines 3, 4, and 5 have active power losses are reduced from 19.7934 kW to 8.2041 kW,from18.5931 kW to 7.1019 kW and from 38.0256 kW to12.1983 kW. This results are less than the base case at normal load i.e. without UPFC device allocation.

Figure 12. Active power losses in each line of IEEE - 33 bus system without and with UPFC allocation at normal load

Figure 13explained thereactive power losses of each line without and with UPFC device on line 5 allocation. This is obvious, line 5 contributes the maximum reactive power loss of the system without UPFC device allocation, followed by line 2. With UPFC device allocation at line 5, there is a noticeable decrease in reactive power loss at each line. The reactive power loss at line 5 has been reduced from32.3250 kVAR to 23.1075 kVAR. The saved reactive power loss at line 5 and line 2 are 9.2175 kVAR and 4.7014 kVAR, respectively when using UPFC device allocation. This results have less than the base case at normal load i.e. without UPFC device allocation.

Figure 13. Reactive power losses in each line of IEEE - 33 bus system without and with UPFC allocation at normal load

At normal load condition, the total active, reactive, and apparent power losses are 111.2288 kW, 108.7720 kVAR and 155.5721 KVA, respectively, while the UPFC system is allocated to line 5. Figure 14 shows that after optimization, the related losses are reduced by 44.82%, 19.15% and 35.82%, respectively.

Figure 14. Total active, reactive and apparent power losses of IEEE 33 bus system without and with UPFC device allocationat normal load

Figure 15 shows the voltage in p.u. at every bus at without and with UPFC allocation between bus 5 and bus 6. From the below Figure it is evident the ability of UPFC device to optimization the voltage profile of a system. Figure 15 indicates that, the voltage range without UPFC device is 0.9134 p.u. to 1.0 p.u. and with UPFC device located at line 5, the voltage range is improving between 0.9583 p.u. to 1.0 p.u.. In addition to the above, the voltage buses near to line 5 are more improving than far busses. It is noticed that before using UPFC device there are 21 buses in total that do not meet the voltage regulation limit but after using the optimization voltages on all buses in this condition are within the voltage regulation constrain.

Figure 15. voltages profile of IEEE - 33 bus system without and with UPFC device allocation at normal load

**5.3 High Load Condition after optimization**

Figure 16 explains the active power losses of each line without and with a UPFC device on line 5 allocation. Line 2 contributes the most to the system's maximum active power loss without UPFC device allocation, as shown in Figure 16 followed by line 5, line 3, and line 4. There is a sharp drop in active power loss from line 1 to line 7 when using the UPFC device allocation at line 5. The other lines after using the optimization from 8 to 32 have the same value or slightly lower of active power loss before using the optimization. The active power loss at high load at line 2 has been reduced from 73.5714 kW to 38.0435 kw after adding the UPFC device allocated in line 5. Other lines 3, 4, and 5 have active power losses are reduced from 28.3929 kW to 11.9792 kW,from26.6071 kW to 10.7350 kW and from 54.8214 kW 21.9900 kW. The percentage reduction of active power losses is 48.29%, 57.81%, 59.65% and 59.88% for lines 2,3,4 and 5, respectively.

Figure 16. Active power losses in each line of IEEE - 33 bus system without and with UPFC allocation at high load

Figure 17discusses thereactive power losses of each line without and with UPFC device on line 5 allocation. This is obvious, line 5 contributes the maximum reactive power loss of the system without UPFC device allocation, followed by line 2. As the UPFC system allocation at line 5 is used, the reactive power loss decreases significantly from line 1 to line 5. The other lines have the same value or slightly reduced reactive power loss after using the optimization from 6 to 32 than before using the optimization. The reactive power loss at line 5 and line 2 have been reduced from46.789 kVAR to 24.8829 kVAR and from30.2655 kVAR to 18.5026 kVAR, respectively. The percentage reduction of reactive power losses is 46.82% and 38.86%, respectively when using UPFC device allocation. This results have less than the base case at high load i.e. without UPFC device allocation.

Figure 17, Reactive power losses in each line of IEEE - 33 bus system without and with UPFC allocation at high load

At high load condition, the total active, reactive, and apparent power losses are 163.7526 kW, 125.4889 kVAR and 202.3065 KVA, respectively, while the UPFC system is allocated to line 5. Figure 18 shows that after optimization, the related losses are reduced by 43.106%, 32.52% and 40.96%, respectively.

Figure 18: Total active, reactive and apparent power losses of IEEE 33 bus system without and with UPFC device allocationat high load

Figure 19 explains the voltage in p.u. at every bus at without and with UPFC allocation between bus 5 and bus 6. Figure 19 demonstrates that, the voltage range without UPFC device is 0.8428 p.u. to 1.0 p.u. and with UPFC device located at line 5, the voltage range is improving between 0.9231 p.u. to 1.0 p.u.. Also it has been discovered that, in addition to the aforementioned, the voltage buses near to line 5 are more improving than far busses. We can note that, before using UPFC device there are 21 buses in total that do not meet the voltage regulation limit and contains 17 buses under value 0.90 p.u. (from bus 8 to bus 18 and from bus 28 to bus 33) and the sag voltages are achieved but after using the optimization, the voltages on all buses in this condition were overcome the voltage sags and 8 busesbecame within voltage regulation constrain.

Figure 19. voltages profile of IEEE - 33 bus system without and with UPFC device allocation at high load

Table 6 tabulates, a comparison of the active power loss, the reactive power loss and voltage profile improvement of the IEEE -33 bus RDS in the absence of a UPFC device and also if the UPFC device is located on line 5 for low, normal and high loads.The results shown in Table 6, proved that with the use of the UPFC device, it is much better when the UPFC device is not used. The percentage reduction of active power losses with UPFC device are 22.16%, 44.82%, and 44.82, while the percentage reductions in reactive power loss are 21.14%, 19.15% and 32.52% for low, normal and high loads, respectively.Also, the minimum voltages are enhanced from 0.9575 p.u. to 0.9695 p.u., from 0.9134 p.u. to 0.9583 p.u. and from 0.8428 p.u. to 0.9231 p.u. for low, normal and high loads, respectively.

Table 6. Comparison between using UPFC device and without UPFC device.

| **Load Condition** | **Parameters** | **Without UPFC** | **With UPFC** |
| --- | --- | --- | --- |
| **Low Load** | Total active power losses (kW) | 54.8465 | 42.6926 |
|  | Total active losses reduction (kW) | 0.0 | 12.1539 |
|  | Percentage active reduction (%) | 0.0 | 22.16 |
|  | Total reactive power losses (kVAR) | 35.0876 | 27.6682 |
|  | Total reactive losses reduction (kVAR) | 0.0 | 7.4194 |
|  | Percentage reactive reduction (%) | 0.0 | 21.14 |
|  | Minimum voltage (p.u.) | 0.9575 | 0.9695 |
|  | Maximum voltage (p.u.) | 1.0 | 1.0 |
|  | Voltage deviation (ΔV) (p.u.) | 0.0425 | 0.035 |
|  | Voltage profile improvement (p.u.) | 0.0 | 0.012 |
| **Normal Load** | Total active power losses (kW) | 201.5943 | 111.2288 |
|  | Total active losses reduction (kW) | 0.0 | 90.3655 |
|  | Percentage active reduction (%) | 0.0 | 44.82 |
|  | Total reactive power losses (kVAR) | 134.5456 | 108.7720 |
|  | Total reactive losses reduction (kVAR) | 0.0 | 25.7736 |
|  | Percentage reactive reduction (%) | 0.0 | 19.15 |
|  | Minimum voltage (p.u.) | 0.9134 | 0.9583 |
|  | Maximum voltage (p.u.) | 1.0 | 1.0 |
|  | Voltage deviation (ΔV) (p.u.) | 0.0866 | 0.0417 |
|  | Voltage profile improvement (p.u.) | 0.0 | 0.0449 |
| **High Load** | Total active power losses (kW) | 287.8240 | 163.7526 |
|  | Total active losses reduction (kW) | 0.0 | 124.0714 |
|  | Percentage active reduction (%) | 0.0 | 43.106 |
|  | Total reactive power losses (kVAR) | 185.9688 | 125.4889 |
|  | Total reactive losses reduction (kVAR) | 0.0 | 60.4799 |
|  | Percentage reactive reduction (%) | 0.0 | 32.52 |
|  | Minimum voltage (p.u.) | 0.8428 | 0.9231 |
|  | Maximum voltage (p.u.) | 1.0 | 1.0 |
|  | Voltage deviation (ΔV) (p.u.) | 0.1572 | 0.0769 |
|  | Voltage profile improvement (p.u.) | 0.0 | 0.0803 |

The performance of the installation UPFC device at line 5 is tested on 33-bus RDS. The proposed PSO algorithms is used to find the optimal allocation for UPFC device to minimize the total power loss with respect the voltage profile within range. The 33-bus RDS consists of 33 buses and 32 lines and the single-line diagram (SLD) of this test system is shown in Figure 6. The line data and load data for this system are in [46]. The base voltage of this system is 12.66 kV and base power is 100 MVA. The system has the total active load is equal to 3715 kW and the total reactive load is equal to 2300 kVAR. The total active and reactive power losses of this system are 201.5943 kW and 134.5456 kVAr, respectively, without installation of UPFC device at normal load condition. To validate the performance of the proposed PSO algorithm, it is applied on this considered test system. The aim here is to, together, minimize total power loss and improve voltage profile. In this case, one number of UPFC device optimally allocated in the radial distribution system. The simulation results for this test system with installation UPFC device are tabulated in Table 6, under the name proposed PSO and are compared with the results obtained by SPSO [47], Firefly-SPSO-SBAT [48], BA with installation DSTATCOM [49], PSO with installation DG [50], EPSO-EP-PSO [51], IA with installation DSTATCOM [52], TLBO- QOTLBO with installation DG [53], GA-AC- IAICA [54], FWA [55], HAS [56] and RGA [57]. From Table 7, it may be noted that of all the algorithms listed, the proposed PSO has the lowest total power loss. The minimum voltage after optimization obtained by the proposed PSO method is much better than the all algorithms indicated in this Table and The system's voltage profile is also improved. Table 7 shows, CPU time for each algorithm. It can be seen that SPSO [47] and SPSO-SBAT [48] have a faster convergence than proposed PSO but the CPU time for proposed PSO is faster convergence than the other methods indicated in the table 7. So, it may be noted that the proposed PSO algorithm better solution than other algorithms in terms of the loss reduction and characteristics of convergence.

Table 7.Comparison of the proposed PSO with other methods on a 33-bus RDS.

| **Method** | **Device /**  **Tie switch** | **Optimal location at line / No.** | **Year** | **Total power loss (kW)** | **Loss reduction (kW)** | **Minimum voltage after optimization (p.u.)** | **Improvement of voltage (p.u.)** | **CPU time**  **(Sec)** |
| --- | --- | --- | --- | --- | --- | --- | --- | --- |
| Proposed PSO | UPFC | 6 | - | **111.2288** | **90.3655** | **0.9583** | **0.0449** | 6.37 |
| SPSO [47] | 33,34,35,36,37 | - | 2020 | 112.58 | 89.0143 | 0.9500 | 0.0366 | **4.02** |
| Firefly [48] | 7,9,14,32,37 | - | 2019 | 142.8 | 58.7943 | 0.93782 | 0.02442 | 8.121 |
| SPSO [48] | 7,9,14,32,37 | - | 2019 | 140.42 | 61.1743 | NM^*^ | NM^*^ | **6.173** |
| SBAT [48] | 7,9,14,32,37 | - | 2019 | 141.20 | 60.3943 | NM^*^ | NM^*^ | **6.025** |
| BA [49] | DSTATCOM | 30 | 2017 | 143.38 | 58.2143 | 0.9260 | 0.0126 | 6.5 |
| BA [49] | DSTATCOM | 11,24,30 | 2017 | 132.08 | 69.5143 | 0.9361 | 0.0227 | 9.62 |
| PSO [50] | DG | 30 | 2015 | 151.38 | 50.2143 | 0.9500 | 0.0366 | NM^*^ |
| EPSO [51] | 11,28,32,34 | - | 2015 | 120.7 | 80.8943 | 0.9980 | 0.006 | 12.2 |
| EP [51] | 17,7,1037,13 | - | 2015 | 125.2 | 76.3943 | 0.9980 | 0.006 | 55 |
| PSO [51] | 7,10,28,14,32 | - | 2015 | 126.4 | 75.1943 | 0.9975 | 0.0055 | 16.0 |
| IA [52] | DSTATCOM | 12 | 2014 | 171.81 | 29.7843 | 0.9258 | 0.0124 | 21.22 |
| TLBO [53] | DG | 14,29,30 | 2014 | 126.496 | 75.0983 | 0.9302 | 0.0168 | 12.64 |
| QOTLBO [53] | DG | 14,27,33 | 2014 | 115.425 | 86.1693 | 0.9324 | 0.019 | 12.58 |
| GA [54] | 7,9,14, 32,37 | - | 2014 | 139.5101 | 62.0842 | 0.9297 | 0.0163 | NM^*^ |
| AC [54] | 7,9,14,28,32 | - | 2014 | 139.9383 | 61.6560 | 0.92977 | 0.01637 | NM^*^ |
| IAICA [54] | 7,9,14, 32,37 | - | 2014 | 139.5101 | 62.0842 | 0.9500 | 0.0366 | NM^*^ |
| FWA [55] | 7,14,9,32,28 | - | 2014 | 139.98 | 61.6143 | 0.9413 | 0.0279 | 6.4 |
| HSA [56] | 7,10,14,37,36 | - | 2011 | 138.067 | 635273 | 0.9342 | 0.0208 | 7.2 |
| RGA [57] | 7,9,14,32,33 | - | 2002 | 139.532 | 62.0623 | 0.9315 | 0.0181 | 13.8 |

**NM^*^**: means not mentioned in the referred reference and the best results are boldly shown in this Table.

**SIMULIK IN MATLAB**

**In a new paper**SIMULIK IN MATLAB was used to study more than one effect on the condition of a real reactor, and the reactor loads were entered into SIMULINK to study more than one effect affecting the reactor.

Effect of UPFC-based PI controller on enhancing active and reactive power flow control

UPFC is used to control either active power only, reactive power only, or both. The following tests are divided as follows:-

a. State 0: Active and reactive power flow signal.

B. Case 1: Both active and reactive power control.

C. Case 2: Maximum effective power control.

D. Case 3: Maximum reactive power control.
